# Neurophysiology of Brain Networks Underlies Symptoms of Parkinson’s Disease: A Basis for Diagnosis and Management

**DOI:** 10.3390/diagnostics13142394

**Published:** 2023-07-18

**Authors:** Martha Teresa Acosta-Mejia, Nelson Villalobos

**Affiliations:** 1 Área Académica de Nutrición, Área Académica de Farmacia, Instituto de Ciencias de la Salud, Universidad Autónoma del Estado de Hidalgo, Ex-Hacienda La Concepción, Sn Agustin Tlaxiaca, Estado de Hidalgo 42160, Mexico; martha_acosta8967@uaeh.edu.mx; 2 Academia de Fisiología, Escuela Superior de Medicina, Instituto Politécnico, Nacional, Plan de San Luis y Díaz Mirón, Colonia Casco de Santo Tomás, Ciudad de Mexico 11340, Mexico; 3 Sección de Estudios de Posgrado e Investigación de la Escuela Superior de Medicina, Instituto Politécnico Nacional, Plan de San Luis y Díaz Mirón, Colonia Casco de Santo Tomás, Ciudad de Mexico 11340, Mexico

**Keywords:** network, neurophysiology, oscillation, basal ganglia, thalamus, dorsal motor nucleus of vagus nerve, symptoms, diagnostic, pathophysiology, beta band

## Abstract

Parkinson’s disease (PD) is one of the leading neurodegenerative disorders. It is considered a movement disorder, although it is accepted that many nonmotor symptoms accompany the classic motor symptoms. PD exhibits heterogeneous and overlaying clinical symptoms, and the overlap of motor and nonmotor symptoms complicates the clinical diagnosis and management. Loss of modulation secondary to the absence of dopamine due to degeneration of the substantia nigra compacta produces changes in firing rates and patterns, oscillatory activity, and higher interneuronal synchronization in the basal ganglia–thalamus–cortex and nigrovagal network involvement in motor and nonmotor symptoms. These neurophysiological changes can be monitored by electrophysiological assessment. The purpose of this review was to summarize the results of neurophysiological changes, especially in the network oscillation in the beta-band level associated with parkinsonism, and to discuss the use of these methods to optimize the diagnosis and management of PD.

## 1. Introduction

Neurodegenerative diseases were one of the first ailments to receive medical attention worldwide [1]. Clinically, these diseases show heterogeneous and overlaying symptoms. Hence, a framework that hones diagnosis will allow better specific treatment and management. Parkinson’s disease (PD) is one of the leading neurodegenerative disorders with heightened prevalence [2,3]. It is considered a movement disorder, although it is accepted that the classic motor symptoms are accompanied by a myriad of nonmotor symptoms, as well as hyposmia, urinary dysfunction, orthostatic hypotension, memory loss, depression, pain, gastrointestinal dysfunction, and sleep disturbances [4]. Gastrointestinal symptoms include drooling, dysphagia, disabled gastric emptying, constipation, and impaired defecation [5,6].

Several cellular mechanisms, including mitochondrial dysfunction, oxidative stress, neuroinflammation, and deficient protein degradation are implicated in the pathogenesis of PD. Nonetheless, the pathological fingerprint consists of neural inclusions of Lewy bodies (LBs) and Lewy neurites, with cell loss in the substantia nigra and other brain areas. The burgeoning of LBs begins from an initial template of alpha-synuclein, which incites the seeding of nearby alpha-synuclein proteins, which triggers the formation of aggregates into a toxic, insoluble pleated sheet structure, to form LBs [7]. At the network neurophysiology level, these pathological fingerprints lead to rearrangement in the electrophysiological and neurophysiological activity that generates the symptoms [8,9,10].

Despite the above, the diagnosis is complicated by the overlap of motor and nonmotor symptoms, in addition to the possibility of other neurodegenerative diseases; as a result, disease management remains suboptimal [4]. In recent years, the criteria for diagnosis have been designed and validated and are dependent on the presence of motor symptoms [4,11,12]. In this context, we reviewed insights related to the neurophysiology of brain networks associated with symptoms of PD and describe evidence from neurophysiological tests that contribute to better diagnosis and management.

## 2. Network Involvement in Motor Symptoms

Motor symptoms dominate the clinical expression of PD. Muscular rigidity, akinesia, bradykinesia, gait instability, and resting tremor form the core of the motor symptoms [9,11,12]. The concept of “parkinsonism” encompasses all motor impairments. For the clinical diagnosis, parkinsonism is defined as bradykinesia accompanied by resting tremor, rigidity, or both [7,11,12]. Dopamine (DA) loss secondary to degeneration of the substantia nigra pars compacta (SNc) initiates parkinsonism by causing impaired modulatory function in the motor network [7,9,13].

**Figure 1:**
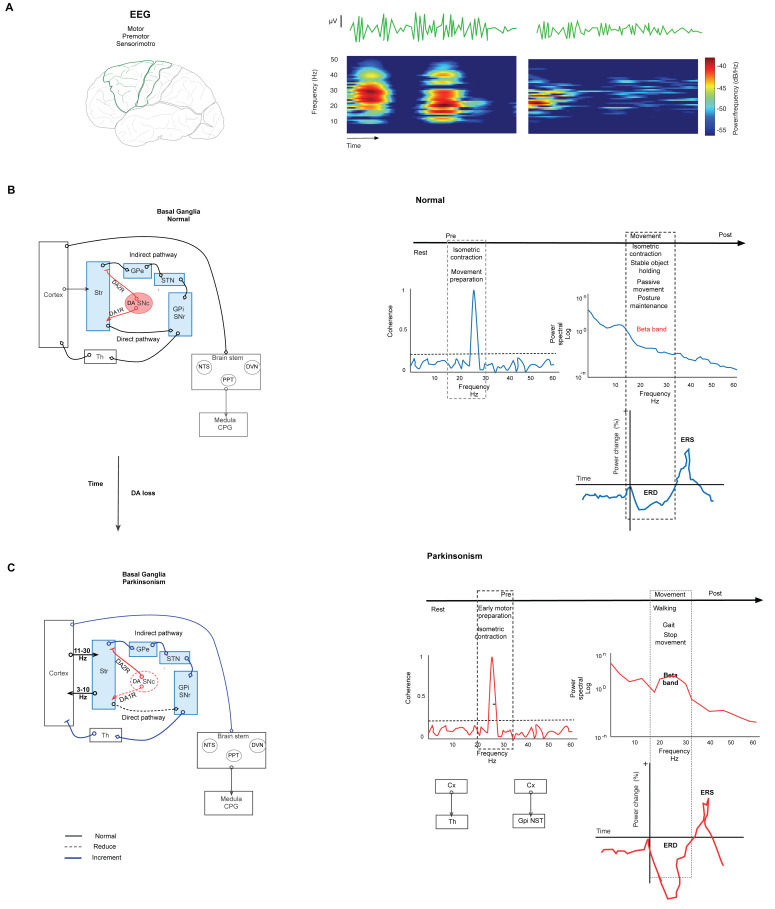
Basal ganglia network in normal and parkinsonism conditions. (**A**) Spectrogram representation of EEG recording of power in beta-band activity in motor and premotor cortex. The spectrogram shows changes in power at the frequency of the beta band. (**B**) Left. Simplified schematic of the BG network in normal conditions. The BG modulate the motor output through the projection neurons of the Str that send their axons to the output nuclei: GPi/SNr in two pathways. The direct path connects the Str with GPi/SNr. In the indirect path, the Str connects with the GPe, the STN, and GPi/SNr. Synthesized by SNc, DA is the network modulator through two types of receptors expressed in both pathways. D1 receptors are expressed in the direct path, and D2 receptors are expressed in the indirect path. Under normal conditions, DA release in the Str reduces the combination of these effects, modulating the GPi and SNr activity, and thus it reduces the inhibition of thalamocortical neurons. Right. Graph representation of changes in the parameters of EEG in normal conditions in several stages of movement. The coherence increments are during isometric contraction and training preparation. Beta power declines during movement performance, postural maintenance, and stable object holding. The same movement events show the desynchronization of beta power (ERD). (**C**) Simplified schematic representation of the BG network in parkinsonism. Left. Degeneration of the nigrostriatal pathway causes DAloss-induced aberrant transmission of the sensorimotor Str and reduces both the direct pathway’s tonic excitation and the indirect pathway’s tonic inhibition; consequently, the BG overinhibit their thalamic and brainstem targets. This causes motor symptoms and aberrant oscillation in beta-band frequency. Right. Changes in beta-band frequency are shown in coherence, power, and ERD during motor performance. The coherence increment is during isometric contraction and movement preparation between CxM-Th and CxM-Gpi/NST. The power density of an ERD shows increment during gait, walking, and movement cessation. Continuous lines represent physiological connections. The dashed line represents a decrease. The bold line represents increased activity. SNc. Substantia nigra compacta. GPi. Globus pallidus internus. GPe. Globus pallidus external. STN. Subthalamic nucleus. Str. Striatum. The graft was elaborated based on the data reported in the main text and supported by the respective references.

Motor output is modulated by the basal ganglia (BG). The BG comprises several nuclei: the striatum (Str), the external (GPe) and internal (GPi) segments of the globus pallidus (GP), the subthalamic nucleus (STN), the substantia nigra compacta (SNc) and the reticulata (SNr). Functionally, the cortex (Cx) sends motor information by excitatory axons to the Str, STN, and thalamus (Th). In this way, the information reaches the circuit through the Str and emerges through the output nuclei, the GPi/SNr, which then send the information to Th.The Str dually organizes the circuits according to the projection neurons that send their axons to the output nuclei. Thus, the connection between the Str and GPi/SNr forms the “direct” pathway. At the same time, the Str establishes a connection, before reaching the output nuclei, with the GPe and the STN, giving rise to the “indirect” pathway (Str-GPe-STN-GPi/SNr; Figure 1B left) [14,15,16]. In turn, this network forms larger parallel circuits that include the frontal Cx, ventral Th, and two nuclei of the brainstem: the superior colliculus [17] and the pedunculopontine nucleus (PPN) [18]. Based on the functions of the cortical area of origin, the BG-Th-Cx network is designated as “motor”, “associative/cognitive”, and “limbic” circuits [8,19]. In our context, parkinsonism arises from abnormal activity patterns in the motor circuit (Figure 1C left).

Synthesized in the SNc, DA is a critical modulator in the network. Expressed in both direct and indirect pathways, DA receptors are coupled to different second messenger systems (through Gs or Golf for D1-like receptors and Gi or Go for D2-like receptors). In the direct pathway, DA acts on D1 receptors to inhibit the BG output nucleus. DA acts on D2 receptors in the indirect pathway to suppress activity. Under normal conditions, DA release in the Str reduces the combination of these effects under GPi and SNr activity, reducing the inhibition of thalamocortical neurons that receive the input from the output nucleus (Figure 1B) [20,21]. Thus, the loss of DA by nigrostriatal pathway degeneration induces aberrant transmission of the sensorimotor striatum [21] (more strongly than transmission to the associative and limbic regions); consequently, this pathway degeneration allows GPe-STN activity to go into overdrive, thus raising the inhibition of STN neurons and their projections to output nuclei [22,23]. This change causes parkinsonism (Figure 1C left).

Neuronal activity patterns play an essential role in determining the integrative functions of the BG. In neural ensembles, information is transmitted through temporal patterns of action potentials [24]. Therefore, it is accepted that the information is encoded in the firing rate of individual neurons [25]. In this context, changes in the firing rate of individual neurons in some specific nuclei of the BG induced by DA loss explain the pathophysiology of parkinsonism. In the network, the loss of DA reduces the direct pathway’s tonic excitation and the indirect pathway’s tonic inhibition [13,21]. Both changes increase the mean firing rates of output nuclei. Consequently, the BG overinhibits their thalamic and brainstem targets [26]. This causes decreased activity in Th and Cx, resulting in akinesia.

Secondary to the loss of DA, the Str and the Th show changes in their firing rate. In the Str, the neurons projecting to the direct pathway show decreased spontaneous activity, while those of the indirect pathway show increased spontaneous activity [8]. In the Th, neurons show a slowed firing rate [27], and their firing is modulated during reaching movements [8]. The GPe shows a decreased firing rate after DA depletion [28], and at the same time, the local levels of GABA are increased [29,30]. Reports of changes in the motor cortex (CxM) in parkinsonism are scarce. Recently, the decreased firing of neurons projecting to the pyramidal tract was shown, but did not affect those projecting to the Str [31]. These results suggest that transmission from cortical neurons to pyramidal tract neurons might be involved in motor symptoms associated with parkinsonism [8]. It has been proposed that cortical firing activity could be secondary to dysfunction in the BG and Th and possible changes in the Cx secondary to the loss of DA and other neurotransmitters [32]. However, other studies show findings related to the alterations associated with parkinsonism, both in firing frequency and patterns and network synchrony.

### Firing Pattern Implication in the Neurophysiology of the BG Network

Physiologically, the action potential is the canonical form of information transmission in the brain. Nevertheless, it is known that specific neurons, based on their intrinsic electrical properties, can show increased mean firing frequency over a short time; this type of activity is described as the burst pattern [13,33].

In the BG normal network, the firing pattern of the GPi, GPe, and STN neurons is random, although action potentials do not occur in bursts. In this condition, the GPi fires action potentials continuously at high frequency. In the same way, the GPe fires action potentials at high frequency, although with pauses, and the STN also fires action potentials continuously but in a medium range of frequencies [13]. These characteristics change considerably secondary to DA depletion. Extensive evidence allows us to accept that DA depletion modifies the intrinsic properties of the neurons of different nuclei of the circuit [34,35,36,37]. Similarly, the GPe shows an increase in the range in which burst firing occurs, adding to the diminished firing rate [38,39]. Similarly, the STN shows a modified firing pattern similar to bursting and increased firing frequency [40]. Notably, burst activity in the STN has been resolutely correlated with clinically severe parkinsonism in patients with PD [41].

The reciprocal connection between the GPe and STN inside the network is physiologically transcendental. Both cores are considered the pacemaker of the network [42], and this notion is given particular importance in the development of bursts [23]. Higher activity from the indirect pathway onto the GPe that guides rhythmicity is demonstrated after DA loss by increased density in the synaptic link between both cores; consequently, the GPe increases inhibition that causes a hyperpolarization-induced higher burst pattern in STN neurons [23,42]. In addition, during parkinsonism, neurons of the GPe and GPi increase synchronization, and the GPi tends to fire in a burst pattern [43].

Thalamic neurons have intrinsic properties that allow them to burst under normal conditions or exhibit tonic bursting depending on the physiological state [44]; the burst pattern trends for the BG nuclei are similar to those in the Th [45], especially in the motor region [40,45]. Notably, burst activity results from a convergence of axons from the cerebellum in the Th motor regions [8]. In this sense, a connection between the cerebellum and BG was described recently and suggested implications for parkinsonism symptoms [8,46]. Therefore, the cerebellum-Th-Cx network contributes to parkinsonism [47].

## 3. Network Oscillation

Burst firing is fundamental in network physiology. This pattern augments the reliability of communication between neurons and contributes to integrating local and distal network information [48]. When bursting occurs rhythmically in an ensemble, it results in oscillatory activity [13]. At the same time, the oscillatory activity that is caused by the temporal interaction of neural activity in the network causes synchronization [49]. As previously described, pathophysiological changes induced by DA loss modify the firing pattern of all nuclei integrated into the BG-Th-Cx network; as a result, abnormal oscillatory activity and dysfunctional synchronization originate [49]. A wide range of current evidence holds that motor output and parkinsonism occur in the beta frequency band [50,51,52,53,54].

In the resting state, the primary CxM, the premotor area, and the cerebellum exhibit widespread oscillatory activity in the beta band (Figure 1A) [55]. Currently, the beta band is described in two subbands: low (13–19 Hz) and high (20–30 Hz). Cortical oscillations induce oscillations in the BG; this functional link leads to the spread of oscillatory behavior inside the Cx-BG-Th network [49,54]. The BG structures associated with oscillations in the beta band at rest are the Str [56], the GPe-STN microcircuit [23], and the STN [57]. A computational modeling study suggested that the NST transmits abnormal beta oscillations initiated in the BG to the CxM and Th. This study highlights the importance of the hyper-direct pathway (CxM-NST) as a driver of the beta oscillation originating from the NST [58]. Interestingly, coherence was demonstrated between the supplementary motor area and STN within the high (21–30 Hz) but not low (13–21 Hz) beta frequency range. In addition, supplementary motor area activity selectively drove STN activity at high beta frequencies, suggesting that high beta frequencies propagate from the cortex to the basal ganglia via the hyper-direct pathway [59]. Importantly, the main oscillatory activity at rest occurs at a very slow time scale [60].

The physiological effect of network synchronization in the motor system was identified during movement kinetics. The parameter analyzed was beta power. In this way, during motor behavior, the beta band displays minimal power in movement stages and high power amid postural maintenance, such as during stable object holding [61,62,63,64]. The motor task is a shared resource for studying beta bands during motor performance. Thus, during movement execution and changes in isometric muscle contraction, beta power is lowest. The drop in beta power during movement is observed bilaterally, sometimes with a contralateral preponderance. A decrease in beta power also occurs when no active muscle contraction is needed, such as during action observation or passive movement (Figure 1B right) [64].

A relative increase in beta-band power occurs during static postural maintenance, such as when keeping an object stable [64,65]. The onset is approximately 300 ms after the beginning of the stable grasp [63]. In the course of static holding, the tonically contracting muscles display significant coherence and phase synchronization in the beta band. Likewise, the firing of cortical motor neurons (including pyramidal tract neurons) is phase-locked to the beta band [64]. Notably, the thalamocortical network displays coherence in the beta band during movement preparation, isometric contraction, and at rest [49,63,66]. Throughout movement kinetics, beta power shows a progressive decline approximately 1–2 s before movement starts. Importantly, this power decrease seems to be specific to limb movement coordination. Conversely, 300 to 1000 ms after movement is completed, beta power exhibits a prominent increase known as postmovement beta rebound [55,64,67].

### Network Oscillation in Parkinsonism

In parkinsonism, the structures that show aberrant oscillation in the beta-band frequency are the Cx, Str, GPe, STN, GPe-STN loop, and output nuclei GPi/SNr. Additionally, cortical coupling occurs at 10–35 Hz, which correlates with parkinsonism-related acerbity [54]. This process diminishes after both DAergic treatment and STN stimulation [54]. The 11–30 Hz frequency range is mainly anti-kinetic [64]. In this sense, synchronized oscillations between the STN and GPi were shown in PD patients in this frequency range [68,69,70,71,72,73,74], which has implications for akinesia or bradykinesia. Notably, the use of beta-band activity can predict bradykinesia of the upper limb [75]. Furthermore, coherence between the CxM and STN-Gpi is displayed in PD patients without pharmacological therapy, and it is reduced after they begin pharmacology therapy and in response to voluntary movement (Figure 1C right) [72].

In contrast, tremors exhibit a low range of oscillatory activity: 3–10 Hz. The nuclei implicated are the GPi, STN, and Th [49]. Notwithstanding this, the network implicated in genesis is not limited to the BG-Th; it also includes the cerebellum-Th-Cx network and the interaction among these regions [47,73]. In this way, oscillatory activity in the CxM is consistent with the oscillations in the Th, BG, and cerebellum; simultaneously, it propagates to the STN and STN–GP networks. Therefore, the primary motor cortex takes part in the network generating tremor.

During choice–reaction tasks, parkinsonism exhibits cortical and subcortical regions that display event-related desynchronized (ERD) activity before and during performance. In addition, during task performance, the moment of early motor preparation presents high beta-band desynchronization, and the high beta band increases in power during the “stop” phase of movement [54,74,76]. Relevantly, in rest and isometric muscular contraction, the sensorimotor Cx displays higher power in the beta band during the early stage of PD (Figure 1C right) [77].

Similarly, the gait associated with parkinsonism shows a change in activity in the beta band. In stepping or gait, beta activity shows alternating suppression in PD patients with respect to the resting state. Based on results in the stepping and walking tasks, an alternating suppression of beta activity during stepping or associated with gait in PD patients, equivalent to that in the resting state, was reported. In addition, cortical activity during effective stepping shows excessively high beta (21–30 Hz) over synchronization compared to healthy controls [78]. Additionally, the movement between cued upper and lower extremities induced greater desynchronization of high beta oscillations (24–31 Hz) modulated by movement. PD patients with akinetic rigidity also exhibit beta desynchronization when walking. During regular walking, the suppression of beta oscillations is related to a pattern of a left–right alternation (Figure 1C right) [79,80,81]. Overall, the previous results show that network dynamics disorders in PD patients are centered around increased beta-band frequency.

## 4. Network Involvement in Gastrointestinal Symptoms

The most dominant nonmotor symptoms of PD are gastrointestinal issues, and the common denominator is dysmotility [82,83,84]. Prevalent symptoms are dysphagia (pharyngeal or esophageal), slowed gastric emptying, decreased frequency of bowel movements, and constipation [82,83]. Due to their prodromal characteristics concerning motor symptoms, these symptoms have received more attention for use as a diagnostic method. In this sense, few neurophysiological approaches have been carried out to monitor the symptom characteristics and implications in managing the disease; most approaches have focused on dysphagia, where exciting results have been reported [85].

**Figure 2:**
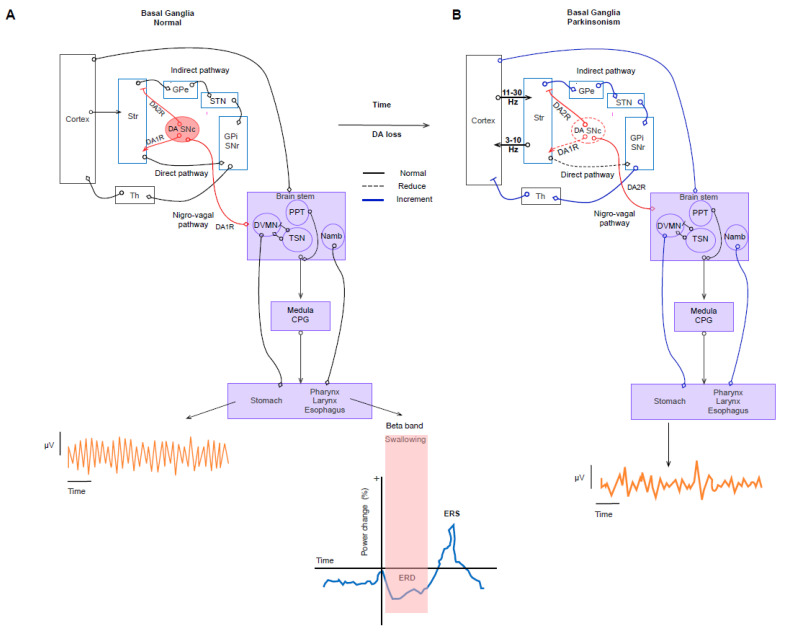
Basal ganglia–brain stem network in normal and parkinsonism conditions. (**A**) Top. Simplified schematic of the BG-brain stem network in normal conditions. A recent anatomical and functional description of the nigro-vagal pathway that influences the vagal output to the upper gastrointestinal tract is included. The DMNV and NAmb originate the vagal efferent that controls the GI. Both nuclei modulated vago-vagal reflexes that mediate both swallowing and gastric emptying. In addition to the PPN, the network modulates the medullar central pattern generator, which regulates the different swallowing phases. Bottom. Left. The drawing represents a typical trace of an electrogastrogram in normal conditions after meal ingestion. A regular amplitude and rhythmicity are observed. Right. The graph represents desynchronization (mu ERD) in the premotor area at the level of the beta band in normal swallowing. (**B**) Simplified schematic of the BG-brain stem network in parkinsonism conditions. Top. Degeneration of the SNc causes DA loss and induces altered modulation of the nigro-vagal pathway into its targets in the brain stem. Consequently, the vagal output to the upper gastrointestinal tract is changed and could be involved in the gastrointestinal symptoms of PD. Bottom. The drawing represents a trace of an electrogastrogram in parkinsonism after meal ingestion. Irregular rhythmicity and decreased amplitude are observed compared with normal conditions. The graft was elaborated based on the data reported in the main text and supported by the respective references.

Neural networks of control for GI physiology embrace four levels. The first level is the myenteric (Auerbach’s) and submucosal (Meissner’s) plexus and enteric glial cells (EGCs). The second level is the prevertebral ganglia, which modulate the peripheral visceral reflex. The third level consists of neurons of the autonomic nervous system (ANS) in the spinal cord originating from the sympathetic (T5L2) and sacral (S2S4) parasympathetic nervous systems. Finally, the fourth level consists of two nuclei of the brainstem: the nucleus tractus solitarius (NTS) and the dorsal motor nucleus of the vagus nerve (DMVN). Both nuclei receive and send signals to the afferent and efferent axons of the vagus nerve (VN), respectively. The DMVN controls the upper GI tract, where myenteric cholinergic neurons mediate the vagal excitatory effect. Pathological alteration of the DMVN occurs in the early stage of PD. Its relevant function forms a substrate that explains GI symptoms that emerge before and overlay motor symptoms (Figure 2A) [84,86].

In addition to the DMNV, the nucleus ambiguus (NAmb) is the origin of the vagal efferent that controls GI function. Therefore, while the DMVN innervates the submucosal and enteric plexuses, the NAmb innervates the striated muscles of the larynx, pharynx, and esophagus. This network controls vago-vagal reflexes that mediate both swallowing and gastric emptying. In addition, the network modulates the medullar central pattern generator, which regulates the different swallowing phases [86,87]. This swallowing network receives axons from premotor, motor, and associative areas of the Cx, BG, and cerebellum. Notably, these cortical areas show activation during swallowing. Regarding oscillation, essential results were recently reported. In the premotor area, desynchronization (mu ERD) at the level of the beta band was described during normal swallowing (Figure 2A) [88].

In motor output, modulation of typical, stereotyped motor automatisms (such as locomotion) is achieved by the pedunculopontine tegmental nucleus (PPN). The PPN connects to the Cx and is reciprocal with the BG. Additionally, cholinergic input is sent to the NTS (this nucleus receives vagal sensory inputs from the GI), a key core of the medullary pattern generator network for swallowing. In this context, the first evidence of an anatomically and functionally monosynaptic nigro-vagal pathway that modulates gastric motility directly was described [89] (Figure 2A).

The pathologic hallmarks of PD are present in the DMNV, sacral parasympathetic nuclei, sympathetic ganglia, ENS in both the Auerbach and Meissner plexuses, submucosal plexus in the stomach, and PPN [83,84,86]. In the NAmb, it spares neurons of the compact portion. In parkinsonism, the intrinsic properties of the DMNV are alternated in the presence of accumulation of alpha-synuclein and the modulation carried out by SNc. The spontaneous activity of the DMNV is tonic and modulated by the NST and SNc. DMNV modulation by the SNc is mainly due to the D1 receptor. In the early stage of parkinsonism, the inhibitory response mediated by the D2 receptor suggests a compensatory upregulation of nigro-vagal inputs [10]. According to this evidence, it is clear that the central nuclei in the vagal microcircuit are altered; therefore, it would be feasible to assume that the physiological synchrony of the circuit is changed and, therefore, is the basis of the common symptoms that accompany PD (Figure 2B). Scarce studies have addressed oscillation analysis at this level.

Dysphagia is present in any stage of PD. The alteration that leads to this symptom can be in the oral, pharyngeal, or esophageal phase. The common findings are poor bolus formation, lingual festination, and repetitive tongue elevation during the oral phase; delay in the swallow reflex, slowing of pharyngeal transit and pooling in the valleculae and pyriform sinuses during the pharyngeal phase; and incomplete relaxation of the upper esophageal sphincter and abnormal esophageal peristalsis and gastroesophageal reflux [84,85,90]. The common anomaly in electromyography (MEG) signals reported in PD is prolonged interval activity between the suprahyoid/submental muscles and the swallowing reaction time [84,90,91]. In this sense, a temporally delayed swallowing reflex was correlated with oropharyngeal freezing [92]. Impairment of the oral and pharyngeal phases of swallowing in PD patients reflects the impaired function of the medullary pattern generator network [84]. This final effector network is affected by SNc loss and regulates effects in all important nuclei, the Cx (premotor, associative area), the BG (Str and GP), the PPN, and the NST.

Esophageal alteration is another prevalent GI symptom in PD. During the early stage, its most common alterations are hypotensive peristalsis, whereas in the later stage, the characteristics are diffuse spasm and multiple contractions [84,90]. The pathophysiological mechanism of esophageal dysmotility could involve the interruption of the vagal motor excitatory pathway. In contrast, the vagal inhibitory pathway is affected by segmental esophageal spasm and achalasia. LB inclusions in the myenteric esophageal plexus have been correlated with achalasia [93].

Delayed gastric emptying that evolves into gastroparesis is the symptom more closely linked to the pharmacological management of PD. Delayed emptying and gastric retention derived from levodopa pharmacokinetics led to a fluctuation in drug response and thus contributed to motor fluctuations in PD [84,90,94,95,96]. The motor control of the gastric muscle is mediated by the vagal efferent coming from the brainstem and intramural microcircuit of the ENS; hence, the interaction between the two structures triggers vago-vagal reflexes that modulate the patterns of regular gastric motor activity. Importantly, the vagus nerve mediates spontaneous coherence between brain activity and gastric slow waves, which is reduced after bilateral vagotomy; in this way, the vagus nerve mediates stomach–brain synchrony [97]. Hence, the pathophysiological mechanism involves an impairment of the vagal excitatory efferent from the DMV and myenteric cholinergic neurons secondary to nigro-vagal degeneration (Figure 2B).

Surprisingly, the electrophysiological approach to this symptomatology has been sporadic and scarce. One approach to utilizing electrogastrographic (EGG) signals to analyze evaluated gastric dysfunction in PD reported a smaller amplitude of gastric activity and a decrease in the frequency of activity after a meal. In this paper, the authors suggested the alteration of the vagus nerve as a possible pathophysiological substrate [98]. Dysrhythmia was significantly associated with a longer duration of disease and treatment [99]. Other findings indicated reduced slow-wave rhythmicity and an impaired postprandial response in gastric myoelectrical activity [94,100]. Untreated patients and patients in the early stage of PD showed irregular waves in EGG signals (Figure 2B) [101].

Constipation is another critical and common nonmotor PD symptom directly associated with disease severity, duration, and predicted swift progression. The characteristic manifestations are disturbed stool consistency and excessive straining. Based on this characteristic, anal sphincter and paradoxical puborectalis contraction dysfunction have been suggested [84,87,102]. Although there is currently no first-line method for its management, the utility of electromyography is accepted. The pathophysiology is based on the deposition of LBs in the ganglion of the myenteric plexus. This translates into increased transit time in the small intestine and proximal colon [84,87]. The loss in modulation secondary to nigro-vagal degeneration, as well as the alteration in transmission in the sacral segment that modulates the defecation reflex, has been postulated as part of the pathophysiology.

## 5. Discussion

In this review, we described the neurophysiological bases that support the alteration of circuits in PD. In summary, the motor circuits involved in both the normal and pathological states share a common denominator: synchronization in the beta band, described as an increase in power. Despite the scarce evidence, we also show that the alteration in GI function shares the same feature.

The diagnostic criteria for PD have been continuously adjusted. Nevertheless, the diagnosis remains complicated due to overlapping symptoms and comorbidities. Consequently, in clinical practice, errors in diagnosis are common [4,103]. With this scenario, physicians rationally apply limited diagnostic tests and thus avoid bias in the differential diagnosis. In addition, these tests follow the profitability and diagnostic performance guidelines dictated by the context [4,103] that once established, the disease management continues on the same path. Therefore, there is a need for a method that helps physicians improve diagnosis and guide clinical management [4,104]. As demonstrated by our previous description, neurophysiological techniques provide information on both the physiology and pathophysiology of movement and visceral motor activity [104,105]. These techniques have the advantage that they are performed in a routine clinical neurophysiology laboratory and can be complemented with other more complex methods, such as imaging or behavioral tests. In this way, EEG emerges as a potential technique with firm parameters. Thus, EEG is a convenient, economical, noninvasive, and widely accessible method to detect early pathophysiological changes during PD [103,104,105,106].

One of the resulting parameters of the EEG is coherence. It is a common mathematical method that quantifies the functional connectivity between brain regions [104,107]. In this way, coherence in the cortical beta band correlates with both the duration and severity of PD and is reduced by DAergic treatment [104]. In addition, it has been shown that coherence characteristics in patients with PD are significantly correlated with the scores on the UPDRS scale and (11C)PE2I DAT PET outcomes, demonstrating its adequate sensitivity [104].

Another of the described parameters is the ERD activity of the beta (13–30 Hz) or mu (8–12 Hz) rhythms of the sensorimotor area (C3/C4). It is an alternative parameter for voluntary movements [108]. Suppression of beta and mu rhythms in the cortical motor area begins approximately 1.5 to 2 s before EMG onset during preparation for a volitional movement [109]. Time–frequency transformations, such as wavelet or short-time Fourier, are used to measure ERD [108]. If the movement uses the voluntary motor system, beta power can be reduced by up to 20–30% compared to the baseline in the preparation stage of movement [110]. The greatest application of EEG is, above all, in motor symptoms. Additionally, upper limb bradykinesia can be predicted by tracking beta activity in the circuit primary Cx–premotor Cx [75]. Thus, the analyses of mu and ERD can be used to study swallowing alterations. However, despite their limited application, ERD assessments can contribute positively to the management of PD [90]. One of the important problems during the management of PD is motor fluctuations due to levodopa treatment. In this sense, the use of the EGG provided important results [111]. Thus, a significant relationship was reported between the pharmacokinetics of levodopa and gastric emptying in patients with PD [94,95].

Bidirectional communication in the gut–brain axis is accepted as the basis of the physiological and pathological state [112]. Communication occurs at the hormonal, immunological, and neural levels [113]. In this context, recent evidence supports the fundamental role of the microbiota in the gut–brain axis both in functional and neurodegenerative diseases [114,115]. In our context, changes in the microbiome are correlated with both motor and nonmotor symptoms [116]. Thus, the gut microbiome and chemical elements in the gut lumen function as stimuli to vagal afferents, and, thus, the vagus could be a direct pathway that connects with higher networks [117]. Based on this context, microbiome studies have been postulated as a biomarker of PD [118]. Nonetheless, the previous description suggests a future perspective that is interesting and has been less explored in our context. The vagus nerve responds to luminal content by increasing its firing rate, and this response’s characteristic depends on the stimulus type [117]. Therefore, neurophysiological parameters could be used to analyze how the changes induced by microbial, nutritional, or pharmacological stimuli transmitted by the vagus nerve to the central network influence the network dynamics that underlie motor and nonmotor symptoms, and thus contribute to better clinical management.

In conclusion, electrophysiological evaluation is a sensitive, noninvasive, and accessible method that can significantly contribute to better diagnosis and management of PD.

## Data Availability

Not applicable.
